# Editorial: Neuromodulation in neurogenic pain and headache

**DOI:** 10.3389/fneur.2025.1645853

**Published:** 2025-07-01

**Authors:** Paweł Sokal

**Affiliations:** Department of Neurosurgery, Functional and Stereotactic Neurosurgery, Collegium Medicum in Bydgoszcz, Nicolaus Copernicus University in Torun, Toruń, Poland

**Keywords:** neurogenic pain, neuromodulation, pain management, headache, neuropathic pain treatment

This section of Frontiers in Neurology provides a dynamic platform where neurologists, pain specialists, anesthesiologists, and neurosurgeons converge to explore innovative strategies for managing neurogenic pain and headaches. The collaborative nature of this forum fosters interdisciplinary insights, ultimately aimed at optimizing patient care.

Migraine, following tension-type headache—which has the highest prevalence at ~26%—is a significantly disabling neurological disorder, affecting nearly 14% of the population. Notably, its incidence is more than three times higher in women than in men. One particularly impactful study in this section suggests that delaying the onset of sexual activity in adolescents may serve as a protective factor against migraine. The authors found a robust causal association between age at first sexual intercourse (AFS) and migraine prevalence. This groundbreaking research highlights the importance of social and behavioral factors in the pathogenesis of migraine. Neuroendocrine, cytokine, and autonomic mechanisms are proposed as plausible mediators of this link, underscoring the biopsychosocial complexity of this relation (Zhu et al.).

Functional neuroimaging studies further deepen our understanding. One notable cohort study revealed altered brain connectivity in patients with migraine with aura. Specifically, these individuals exhibited reduced functional connectivity between the default mode network and the subgenual anterior cingulate cortex, along with increased activity in the supracallosal anterior cingulate gyrus, compared to healthy controls. These findings suggest also disrupted emotional and visual network integration in patients with migraine with aura (Cheng et al.).

Another intriguing investigation focused on breast cancer patients experiencing chronic neuropathic pain and comorbid depression. Resting-state functional MRI revealed significant changes in brain connectivity, linking affective and sensory processing pathways—reflecting the interplay between chronic pain and mood disorders (Liu R. et al.).

Recent advances in molecular research have led to promising therapeutic avenues, particularly the use of monoclonal antibodies targeting key inflammatory pathways. The trigemino-vascular origin of migraine is increasingly associated with genetic predispositions, including elevated levels of NLRP3 and MMP9 inflammasome components in these patients. These biomarkers present a compelling rationale for immunomodulatory therapies aimed at reducing neuroinflammation (Rushendran et al.).

Clinical case reports continue to offer valuable insights. An exceptional case of nummular headache—a localized, coin-shaped scalp pain syndrome—was successfully managed using botulinum toxin therapy, complemented by a ketogenic diet. This dual-modality approach illustrates the potential of integrating pharmacological and dietary interventions (Tereshko et al.).

In contrast, a case series exploring cranial electrical stimulation (CES) combined with transcutaneous electrical nerve stimulation (TENS) for burning mouth syndrome did not yield significant clinical benefits. Despite the use of advanced neuromodulation techniques, results were comparable to placebo, emphasizing the need for more refined therapeutic strategies. The study also underscored the importance of comprehensive outcome assessments, employing tools such as the Pittsburgh Sleep Quality Index (PSQI), Oral Health Impact Profile (OHIP-14), and multiple psychiatric scales (PHQ-D, HAMD, HAMA, HADS) to capture the full spectrum of patient experience. The team from Rostock exemplified rigorous clinical trial design aimed at minimizing placebo effects in neuromodulation research (Palmer et al.).

The integration of vagus nerve stimulation (VNS), a neuromodulation technique, into clinical practice for the management of neurological and psychiatric disorders—particularly chronic pain represents a promising development. A well-defined protocol for a systematic review and meta-analysis assessing the efficacy and acceptability of VNS in fibromyalgia has been presented and is both timely and compelling (Cai et al.).

Cervicogenic headache (CH), which affects up to 20% of patients with chronic headaches, continues to receive growing attention due to its impact on daily functioning. As literature on CH expands, further research is essential to elucidate pathophysiological mechanisms and refine treatment protocols (Xu et al.).

A Chinese research group proposed an innovative treatment for CH involving fluoroscopically guided meridian sinew Tuina injections targeting the occipital nerves. Their randomized controlled trial (RCT) protocol represents an excellent example of integrating traditional Chinese medicine with available imaging techniques (Huang et al.).

The burden of neuropathic pain accompanied by depression presents a significant clinical challenge. A prospective 3-month study evaluated the efficacy of vortioxetine, a multimodal serotonergic antidepressant, in patients with painful polyneuropathy due to entrapment or metabolic disturbances. Results indicated both analgesic and antidepressant benefits, with minimal adverse effects. The drug's potential to enhance neuroplasticity adds a promising dimension to its therapeutic profile (Eliaçık and Erdogan Kaya).

Management of acute herpetic neuralgia and postherpetic neuralgia remains a critical concern for neurologists and pain specialists. Conservative approaches using lidocaine patches, pregabalin, and gabapentin are first-line treatments. However, pulsed radiofrequency thermocoagulation of thoracic dorsal root ganglia represents a minimally invasive alternative aimed at modulating central sensitization with satisfactory effects (Wang et al.).

For patients with refractory trigeminal neuralgia and atypical facial pain unresponsive to pharmacotherapy, deep brain stimulation (DBS) has emerged as a viable solution. Targeting the periventricular and periaqueductal gray regions of the thalamus, DBS achieves nearly 50% pain reduction at 1-year follow-up by modulating ascending nociceptive pathways (Mandat et al.).

In terminal patients with pancreatic cancer experiencing mixed nociceptive and neurogenic pain, CT-guided chemical neurolysis of the celiac plexus has shown to reduce opioid consumption and improve quality of life, albeit without affecting survival. This technique should be incorporated as a core element of comprehensive palliative care strategies (Lu et al.).

Finally, non-pharmacological interventions remain essential in chronic pain management. Exercise has been extensively documented to promote neuroplasticity, enhance circulation, improve muscle function, and reduce inflammation. A meta-analysis by a Chinese group demonstrated that physical activity significantly raises both thermal and mechanical pain thresholds, supporting its inclusion as a standard component in neuropathic pain rehabilitation protocols (Liu S. et al.).

In conclusion, this section highlights the multifaceted nature of neurogenic pain and headache disorders. The diversity of contributions- ranging from molecular research and neuroimaging to case reports and clinical trials—demonstrates the field's rapid evolution. This collaborative platform highlights evidence-based, multidisciplinary strategies for pain management, with a specific emphasis on the advancing field of neuromodulation.

